# Aging-caused the changes of the gut microbiota drive intestinal barrier dysfunction and increase sepsis susceptibility

**DOI:** 10.1080/19490976.2026.2630475

**Published:** 2026-02-21

**Authors:** Huoyan Liang, Xianfei Ding, Shaohua Liu, Shuai Tong, Xu Wang, Zihao Zhang, Wei Wang, Xiaojuan Zhang, Yangyang Yuan, Yong Jiang, Tongwen Sun

**Affiliations:** a Department of Critical Care Medicine, Department of Emergency Medicine, The First Affiliated Hospital of Zhengzhou University, Henan Engineering Research Center for Critical Care Medicine, Henan Key Laboratory of Critical Care Medicine, Henan Key Laboratory of Sepsis in Health Commission, Zhengzhou Key Laboratory of Sepsis, Henan Sepsis Diagnosis and Treatment Center, Zhengzhou, People's Republic of China; b Department of Pediatrics, The First Affiliated Hospital of Zhengzhou University, Zhengzhou, People's Republic of China; c Department of Emergency Medicine, State Key Laboratory of Metabolic Dysregulation & Prevention and Treatment of Esophageal Cancer, Henan Key Laboratory of Critical Care Medicine, Henan International Joint Laboratory of Infection and Immunology, Henan Province Engineering Technology Research Center of Precision Medicine for Organ Dysfunction, The First Affiliated Hospital, Zhengzhou University, Zhengzhou, People's Republic of China; d Institute of Infection and Immunity, Henan Academy of Innovations in Medical Science, Zhengzhou, People's Republic of China

**Keywords:** Sepsis, aging, *K. aero*, histamine, intestinal barrier dysfunction

## Abstract

Physiological and pathological changes associated with aging contribute to deteriorating disease prognosis in sepsis. However, the mechanisms by which these disturbances exacerbate inflammation remain underexplored. In this study, fecal samples were collected from aged and young septic patients and mice and subsequently transplanted into young pseudo-germ-free mice via fecal microbiota transplantation. Fecal, colon tissue, and blood samples were collected to be used 16S rDNA sequencing to characterize the gut microbiota, histopathological examination, enzyme-linked immunosorbent assay and FITC-dextran intestinal permeability assay to assess gut injury and gut barrier function. Additionally, nontargeted and targeted metabolomics were used to identify differential metabolites in the feces of aged and young septic mice. To further validate the roles of specific bacterial strains and their metabolites in sepsis, genetically engineered bacteria were used in both *in vivo* and *in vitro* experiments. The results showed that an increased abundance of *Klebsiella aerogenes* (*K. aero*) in aged hosts, which led to elevated histamine (HA) production and exacerbated intestinal barrier dysfunction. Importantly, *K. aero* strains carrying a histidine decarboxylase gene variant were identified as major HA producers. Mechanistically, HA was shown to drive intestinal barrier dysfunction by inhibiting Nlrp6 expression and its subsequent binding to LC3, thereby impairing autophagy. Treatments that modulated HA levels or overexpressed Nlrp6 ameliorated inflammation in septic mice. These findings suggest that targeting the HA-Nlrp6-LC3 axis could offer a novel therapeutic approach for managing sepsis, particularly in aged populations.

## Graphical abstract

**Figure:**
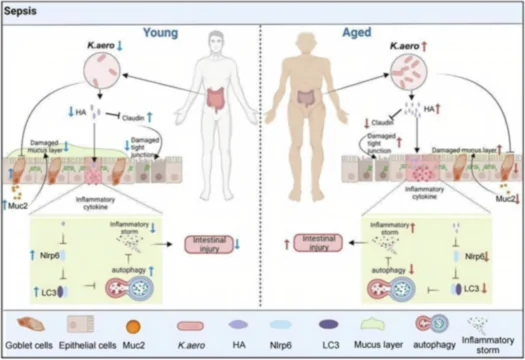

## Introduction

Sepsis, a multifactorial disease resulting from an immune response dysregulation to bacterial infections, is associated with substantial mortality.^1^ In 2017, approximately 48.9 million new cases of sepsis were documented globally, with 11.0 million related deaths, accounting for 19.7% of all worldwide fatalities.^2^ Epidemiological data^3^ indicate that sepsis, severe sepsis, and septic shock accounted for 3.10%, 43.6%, and 53.3% of cases, respectively, with corresponding 90-d mortality rates of 2.78%, 17.69%, and 51.94%. Age is often considered an independent risk factor for mortality and morbidity in sepsis^4^; however, the mechanisms underlying the increased mortality in aged patients remain poorly understood, as do effective therapeutic strategies for this group. Recent studies have shown that 57.5% of sepsis incidents occur in individuals aged over 65 y,^5^ and the incidence rate among the very elderly is 31 times higher than that in adults.^6^ Additionally, septic patients over 65 y old are more likely to experience persistent organ dysfunction, with a twofold increase in 1-y mortality.^7^ Elucidating the mechanisms for the vulnerability of intestinal barrier in aged septic patients may uncover novel therapeutic strategies to enhance clinical outcomes in this high-risk population.

Recent evidence suggests that the gut microbiota is intricately linked to the host life cycle and disease regulation.^8^ Numerous studies have identified a causal relationship between gut microbiota and sepsis-related mortality,^9^^,^^10^ with aging contributing to an increased burden of enterobacterial virulence factors, thereby accelerating the progression of sepsis.^11^ Crucially, the mechanisms through which gut microbiota influence disease pathogenesis include compromised intestinal integrity and the production of metabolites.^12-14^ Our previous research has demonstrated that aging exacerbates dysbiosis of gut microbiota and liver damage in septic mice.^15^ Furthermore, specific strains of the gut microbiota, particularly *Klebsiella* and *Escherichia_Shigella*, have been shown to cause sepsis-related liver injuries in aged rats.^16^ Aging significantly alters the gut microbiota profile, leading to an overabundance of genomic virulence factors that heighten susceptibility to metabolic syndrome.^11^ Consequently, we hypothesize that the increased mortality observed in aged sepsis patients is due to fecal microbiota dysbiosis.

The NOD-like receptor family pyrin domain-containing 6 (Nlrp6) inflammasome, an intracellular sensor of the innate immune system, plays a key role in the colonic mucosal defense against bacterial pathogens^17^^,^^18^ and modulates inflammation by inhibiting the mitogen-activated protein kinase (MAPK) and nuclear factor-κB (NF-κB) signaling pathways.^19^ Additionally, the combination of Nlrp6 and estrogen receptor beta (ERβ) activates autophagy, which is crucial for maintaining homeostasis of intestinal epithelial cells and promoting tissue repair in colitis.^20^ However, the role of Nlrp6 in the pathogenesis of sepsis remains poorly understood.

The purpose of this study was to investigate the impact of intestinal microbiota imbalance on the compromised intestinal barrier in aged sepsis patients. We used homologous recombination of *Klebsiella aerogenes* (*K. aero*) to explore the potential mechanisms underlying the increased production of histamine (HA) in the gut microbiota of the aged population. The disruption of intestinal microbiota due to aging contributes to intestinal barrier dysfunction, thereby enhancing susceptibility to sepsis. This finding underscores potential therapeutic approaches to mitigate the severity of sepsis.

## Results

### Aging worsens septic prognosis

**Figure 1.**
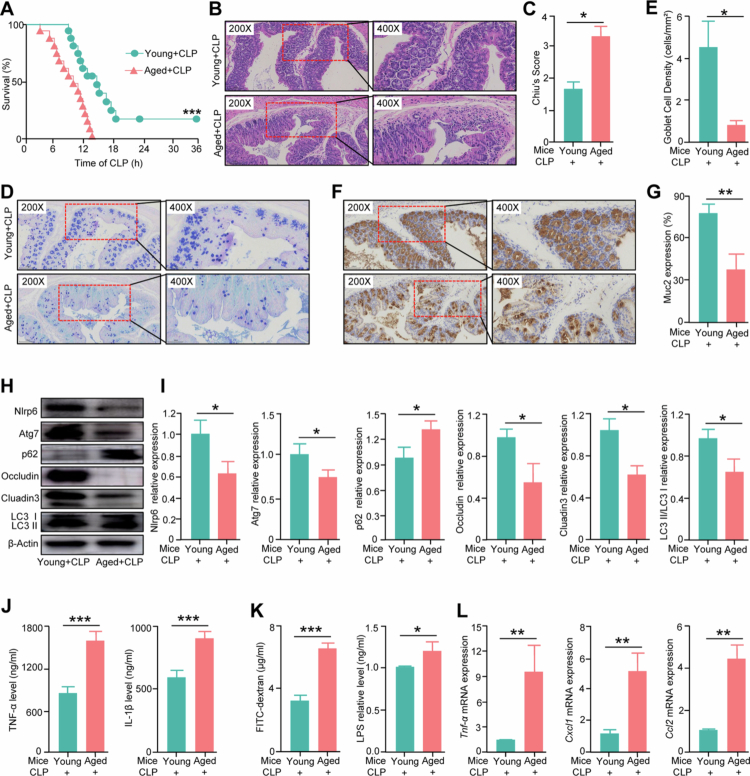
Ageing worsens septic prognosis. (A) The survival rate in young and aged septic mice. *n* = 15, ***, p < 0.001. (B,C) H&E assay (B) and histopathological score (C) in the colon from young and aged septic mice. *n* = 5. *, *p* < 0.05. (D,E) AB‒PAS staining (D) and goblet cell density (E) of colon from young and aged septic mice. *n* = 5. *, *p* < 0.05. (F,G) Immunohistochemistry (F) and quantification of Muc2 expression (G) of colon from young and aged septic mice. *n* = 5. **, *p* < 0.01. (H) Western blot analysis for the quantification of protein expression of colon from young and aged septic mice. *n* = 5–6. (I) Semi-quantitative analysis results of proteins of colon from young and aged septic mice. *n* = 5–6. *, *p* < 0.05; **, *p* < 0.01. (J) Quantification of cytokine levels in the serum of young and aged septic mice. *n* = 4. ***, *p* < 0.001. (K) Serum FITC and LPS levels from young and aged septic mice. *n* = 4. *, *p* < 0.05; ***, *p* < 0.001. (L) Quantification of cytokine mRNA levels in the colon of young and aged septic mice. *n* = 4–8. **, *p* < 0.01.

To determine whether aging increases susceptibility to sepsis, the mortality rates within 36 h following cecal ligation and puncture (CLP) between aged (22–24 months) and young (8–10 weeks) mice were compared. Results indicated a significant increase in mortality in the aged group compared to the young group (Figure 1A). Specifically, about 50% of the young mice died from sepsis within 15 h after CLP, whereas all aged mice succumbed to the condition. Furthermore, to determine whether aging further exacerbates sepsis-induced intestinal barrier dysfunction, we performed histopathological assays, immunohistochemistry, Western blot analysis, and enzyme-linked immunosorbent assay (ELISA). The results showed that that aged septic mice exhibited more severe intestinal injuries (Figure 1B−C), reduced numbers of goblet cells (Figure 1D−E), and decreased Muc2 expression (Figure 1F−G) compared to young mice. Post-CLP analysis showed reduced expression levels of Nlrp6, Occludin, Claudin3, and autophagy-related proteins (Atg7 and LC3II/LC3I) in the aged group (Figure 1H−1I). Additionally, the serum concentrations of TNF-α and IL-1β were also higher in the aged group (Figure 1J). Similarly, intestinal permeability assays indicated higher serum levels of fluorescein isothiocyanate (FITC) and lipopolysaccharide (LPS) in the aged group than in the young group (Figure 1K). Quantitative reverse transcriptase polymerase chain reaction (qRT‒PCR) analyzes of colon tissues showed increased mRNA expression of *Tnf-α*, *Cxcl1*, and *Ccl2* in aged septic mice (Figure 1L). These findings collectively suggest that aging enhances susceptibility to sepsis by exacerbating intestinal injury and barrier dysfunction, accompanied by an intensified inflammatory response.

Subsequently, we compared inflammatory markers and intestinal injury parameters between aged and young sham-operated mice to determine whether the worse outcomes of sepsis in aged individuals result from intrinsic aging effects or age-specific responses during sepsis. While some inflammatory markers were elevated in aged mice, indicators of intestinal damage and barrier function showed no differences between the groups (Figure S1), which suggests that the exacerbation of sepsis outcomes in aging may primarily stem from age-specific pathological mechanisms during sepsis progression. We hypothesize that this phenomenon could be linked to gut microbiota dysbiosis, though further validation is needed.

### Aged septic hosts exhibit higher mortality rates linked to gut microbiota

Intestinal microbiota have been implicated in the pathogenesis of sepsis, and their composition is altered by aging.^21^ To investigate whether changes in the gut microbiota could directly contribute to increased sepsis susceptibility in aged individuals, cecal contents (CCs) from both aged and young mice were collected for analysis. Intraperitoneal injections of these CCs into young mice demonstrated similar sepsis susceptibility across both groups (Figure S2A‒S2D). Subsequently, we eradicated gut microbiota by administering broad-spectrum antibiotics (ABX) for 3 d prior to treatment with CLP based on the previous studies.^22^^,^^23^ The survival rates of aged and young septic mice with ABX pretreatment were comparable (Figure S2E). Additionally, the results revealed no significant differences in histopathological scores, numbers of goblet cells and Muc2 expression (Figure S2F–S2K) compared to young septic mice. Similarly, serum levels of TNF-α and the mRNA expression of *Tnf-α* and *Ccl2* in the colon tissues showed no differences between the young and aged septic mice with ABX pretreatment (Figure S2L−2M). Consistent with the septic mice subjected to ABX pretreatment, both aged and young mice of sham groups exhibited a similarity on intestinal damage and inflammatory cytokines after pretreatment with ABX (Figure S2N−2U).

**Figure 2.**
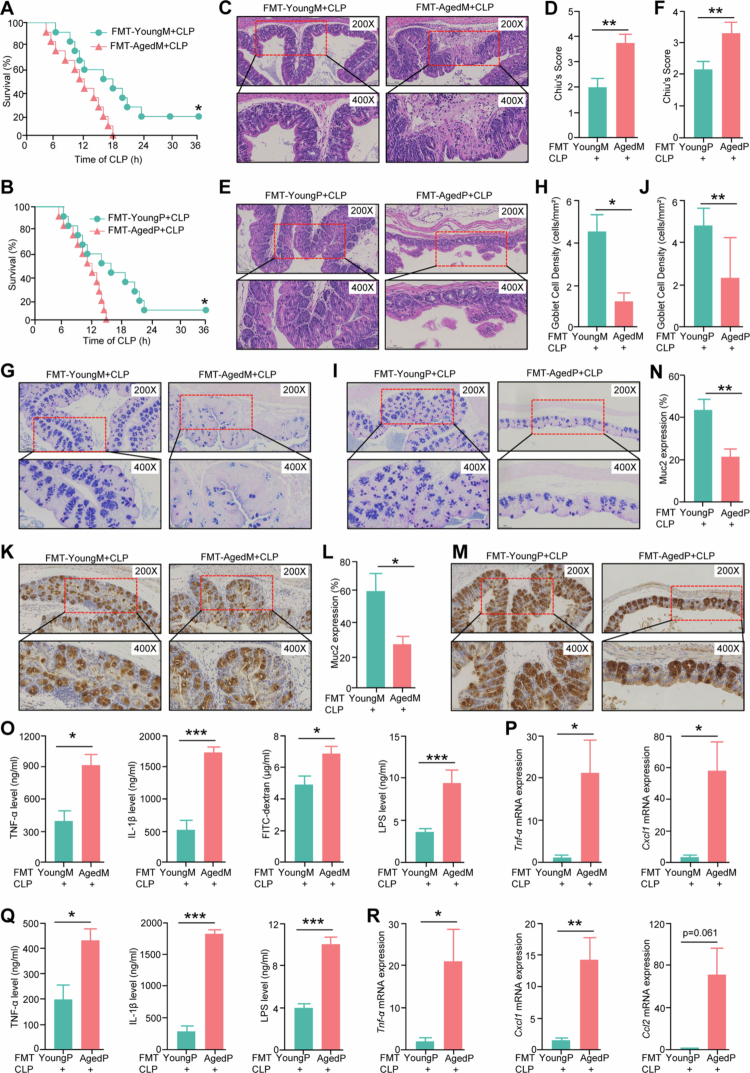
Aged septic hosts exhibit higher mortality rates linked to fecal microbiota. (A,B) The survival rate for the 12 young septic mice per group received feces from young and aged septic mice (A) or patients (B), respectively. *n* = 12 mice/group. *, *p* < 0.05. (C,D) H&E assays (C) and histopathological scores (D) in the colon from the young septic mice received feces from young and aged septic mice. *n* = 5. **, *p* < 0.01. (E,F) H&E assays (E) and histopathological scores (F) of the colon from the young patients feces recipients and aged patients feces recipients. *n* = 4. **, *p* < 0.01. (G,H) AB‒PAS staining (G) and goblet cell density (H) of colon from the young septic mice received feces from young and aged septic mice. *n* = 4. *, *p* < 0.05. (I,J) AB‒PAS staining (I) and goblet cell density (J) of colons from the young septic mice received feces from young and aged septic patients. *n* = 5. **, *p* < 0.01. (K,L) Immunohistochemistry (K) and quantification of Muc2 expression (L) of from the young septic mice received feces from young and aged septic mice. *n* = 4. *, *p* < 0.05. (M,N) Immunohistochemistry (M) and quantification of Muc2 expression (N) of from the young septic mice that received feces from young and aged septic patients. *n* = 5. **, *p* < 0.01. (O,P) Quantification of cytokines, FITC and LPS in the serum, and the cytokines mRNA expression in the colon of the young septic mice received feces from young and aged septic mice. *n* = 4. *, *p* < 0.05; ***, *p* < 0.001. (Q,R) Quantification of serum cytokines, LPS levels, and colonic cytokine mRNA expression in young mice transplanted with fecal suspensions from young and aged septic patients. *n* = 4–7. *, *p* < 0.05; **, *p* < 0.01.

To investigate the impact of intestinal dysbiosis on sepsis progression in aging, we first performed fecal microbiota transplantation (FMT) using donor specimens from aged mice and human patients into the young mice of pseudo-germ-free (PGF) treatment by ABX. To further confirm the reproducibility of our FMT methodology, we subsequently recollected fecal specimens from both young and aged septic mice, as well as from patients, and carried out an additional round of FMT experiments, which further substantiate the efficacy of the approach. The results demonstrated that microbial communities from both donor septic mice (Figure S3A−3B) and patients (Figure S3C−3D) were capable of robust engraftment in the recipient PGF mice. The survival analysis showed that young mice receiving feces from young donors exhibited greater resistance to sepsis compared to those receiving feces from older donors, regardless of whether the fecal source was mice or patients (Figure 2A−2B). Histopathological examinations revealed that young mice and patients gavaged with feces from aged mice developed more severe intestinal injuries (Figure 2C−2F), reduced numbers of goblet cells (Figure 2G−2J), and decreased Muc2 expression (Figure 2K−2N) in response to sepsis than those gavaged with feces from young mice. Additionally, the increased serum cytokine levels and the elevated expressions of cytokines and chemokines in colon tissues from mice that received feces from aged septic mice compared to those that received feces from young septic mice following CLP treatment, and aged feces recipients exhibited higher serum FITC and LPS compared to young feces recipients (Figure 2O−2P). Consistently, recipients of aged septic patients feces exhibited higher LPS and cytokine levels in response to sepsis (Figure 2Q−2R). Collectively, these findings suggest that microbiome alterations associated with aging significantly increase susceptibility to sepsis.

### Aging induces the increased abundances of *Klebsiella* and HA

**Figure 3.**
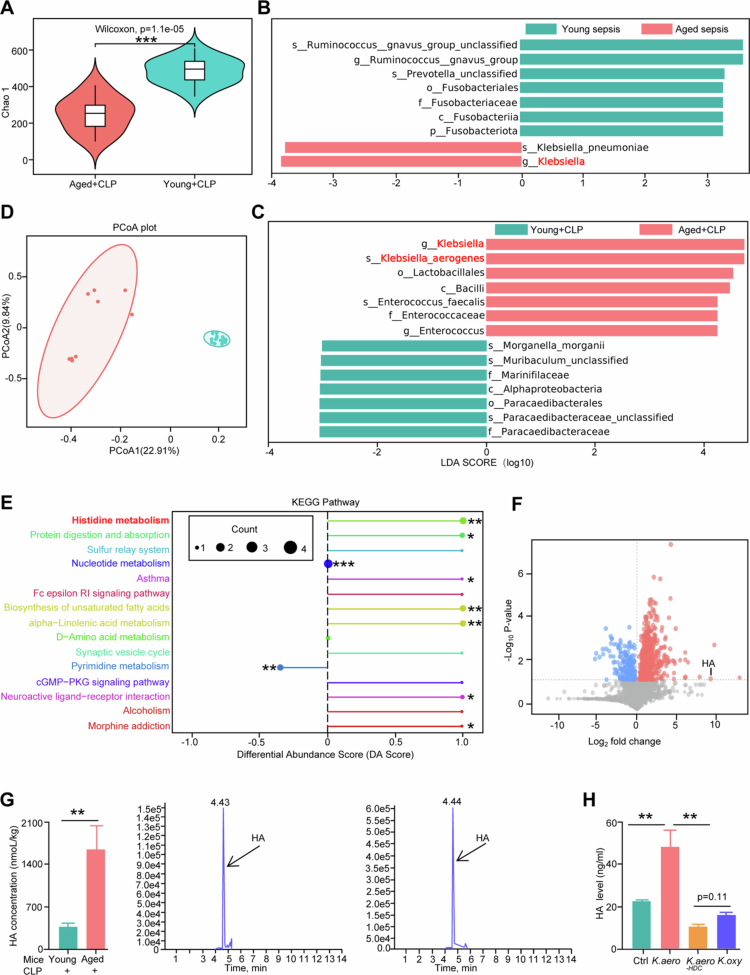
Aging induces the increased abundances of *Klebsiella* and HA. (A) Alpha diversity based on Chao1 in young and aged mice. Young mice, *n* = 15; aged mice, *n* = 9. ***, *p* < 0.001. (B,C) The differences of gut microbiota composition of young and aged septic patients (B) or mice (C) were evaluated by linear discriminant analysis effect size (LEfSe). For young septic patients, *n* = 13; aged septic patients, *n* = 12; for mice, *n* = 9–15. (D) Using the Bray–Curtis distance by principal coordinate analysis (PCoA) in young and aged septic mice. *n* = 9–15. (E) The enrichment of differential metabolites in KEGG pathways. *, *p* < 0.05; **, *p* < 0.01; ***, *p* < 0.001. (F) The volcano plot of gut metabolites from young and aged septic mice. *n* = 8–9. (G) Targeted metabolomics detects the concentrations of HA in cecum content from young and aged septic mice. *n* = 6. **, *p* < 0.01. (H) HA concentration in serum of PBS, wild-type, *K. oxy*, or *HDC*-deficient mutant of *K. aero* strain treated mice subjected to CLP. *n* = 6-8. **, *p* < 0.01.

We investigated the potential mechanisms by which changes in microbiota during aging increase susceptibility to sepsis. 16S rDNA sequencing revealed that the aged group exhibited distinct alpha diversity and marked intergroup separation in gut microbiota composition (Figure 3A). Alpha and beta diversities were similar between aged and young patients (Figure S4A - S4B). Additionally, aging led to alterations in the genus-level composition of intestinal microbiota in patients (Figure S4C). Linear discriminant analysis (LDA) indicated a higher abundance of *Klebsiella* in aged septic patients and mice (Figure 3B−3C). To explore the impact of aging on the metabolic profile of intestinal microbiota, metabonomic analyzes were conducted, revealing significant shifts in fecal microbiota metabolic formations in aged septic mice, as demonstrated by principal coordinate analysis (PCoA) (Figure 3D). The enrichment analysis revealed that differential metabolites were predominantly involved in histidine metabolism (Figure 3E). Given that histamine is the principal metabolic product of histidine and its established role in immune regulation, we selected HA as our primary target. This choice was further supported by volcano plot (Figure 3F) and heatmap analyzes (Figure S4D) showing increased HA levels in aged septic mice. Subsequent targeted LC‒MS/MS quantification confirmed significantly higher HA concentrations in aged versus young hosts (Figure 3G), suggesting aging potentiates HA accumulation during sepsis.

### Histidine decarboxylase from *K. aero* is a key contributor to HA in the intestine

Upon confirming the concurrent increase in *Klebsiella* and HA in aged septic hosts, we observed a significant rise in *K. aero* abundance in these patients (Figure S4E). We measured the relative abundance of *K. aero* in fecal samples from aged and young septic patients and found that the relative levels of *K. aero* were significantly higher in aged patients compared to the young counterparts (Figure S4E). Subsequently, *in vitro* experiments revealed that HA concentrations in the culture supernatant of *K. aero* were higher compared to those in the control culture medium (Figure S4F). Given the high variability of the histidine decarboxylase (HDC) gene in Gram-negative bacteria,^24^ we aimed to further clarify the mechanism of HA production. We assessed HA levels in *Klebsiella oxytoca* (*K. oxy*), *K*. *aero*, a *K. aero* isolate engineered to lack the *HDC* gene (*K. aero*^-HDC^), and control group mice following CLP treatment. The results indicated that only *K. aero* could produce significant levels of HA, whereas *K. oxy*, *K. aero*^-HDC^, and control groups exhibited comparable HA levels (Figure 3H). Additionally, *in vitro* deletion of the *HDC* gene in *K. aero* strains effectively inhibited HA production in the supernatants (Figure S4G). Corresponding with the increased presence of *K. aero*, aging also led to elevated serum HA concentrations in septic mice (Figure S4H), suggesting a critical role for *K. aero* in the progression of sepsis through enhanced HA production during aging.

### Treatment with K. aero or HA worsens inflammation and intestinal injury in septic mice

**Figure 4.**
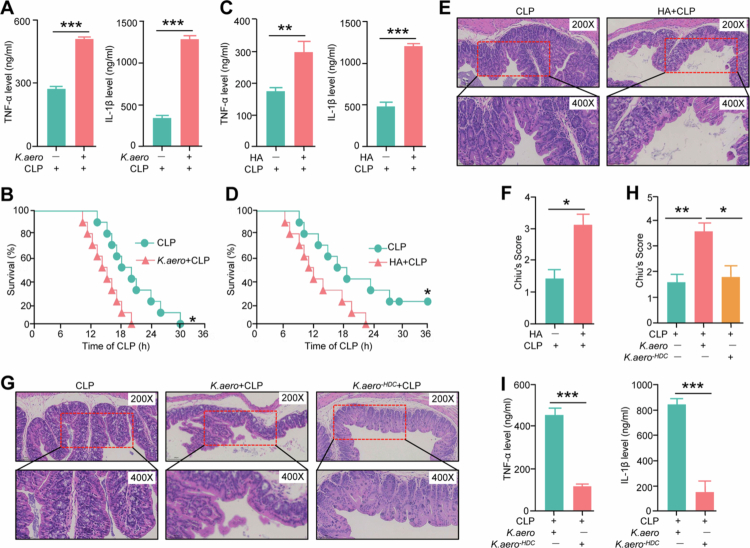
Treatment with *K. aero* or HA worsens inflammation and intestinal injury in septic mice. (A) Cytokines levels in the serum from CLP mice received with or without *K. aero*. *n* = 4. ***, *p* < 0.001. (B) The survival rate of young septic mice pretreatment with *K. aero*. *n* = 10. *, *p* < 0.05. (C) Cytokine levels in the serum from CLP mice received with or without HA. *n* = 4. **, *p* < 0.01; ***, *p* < 0.001. (D) The survival rate of young septic mice pretreatment with HA. *n* = 10. *, *p* < 0.05. (E,F) H&E staining (E) and histopathological score (F) of the colon from young septic mice pretreatment with HA. *n* = 4. *, *p* < 0.05. (G,H) H&E staining (G) and histopathological score (H) of the colon from young septic mice pretreatment with *HDC*-deficient mutant of *K. aero* strain culture supernatants from LB and *K. aero* strains. *n* = 5. *, *p* < 0.05; **, *p* < 0.01. (I) Serum TNF-α and IL-1β levels from young septic mice pretreatment with *K.aero* *and HDC-deficient mutant of K. aero strain. n* = 5. ***, *p* < 0.001.

To assess the influence of *K. aero* on sepsis, cytokine levels were measured using ELISA. The results showed that young septic mice pretreated with *K. aero* exhibited higher serum levels of TNF-α and IL-1β compared to those not receiving *K. aero* (Figure 4A). Similarly, *K. aero* pretreatment was associated with decreased survival rates in aged septic mice (Figure 4B). Consequently, the proinflammatory role of *K. aero* during sepsis was further evaluated to determine its association with HA production. As anticipated, HA elevated the serum concentrations of TNF-α and IL-1β, as well as decreased survival rates in septic mice (Figure 4C−4D). Histological assays indicated that HA treatment exacerbated pathological changes in the colon of septic mice (Figure 4E−4F). Immunohistochemical analyzes showed that HA treatment reduced Muc2 expression and goblet cell numbers in the colon (Figure S5A–S5D). Furthermore, deletion of the *HDC* gene in *K. aero* strains alleviated its detrimental effects on pathological changes in the colon and inflammatory response in septic mice (Figure 4G−4I). Collectively, these findings suggest that both *K. aero* and HA exacerbate the effects of sepsis, influencing inflammatory responses and survival outcomes.

### HA inhibits Nlrp6 expression to exacerbate intestinal barrier dysfunction in septic mice

Increased levels of *K. aero* and its metabolite HA during aging are associated with impaired intestinal autophagy, compromised barrier function, elevated inflammatory cytokine levels, increased gut microbiota translocation, and heightened susceptibility to sepsis. To elucidate how HA contributes to increased susceptibility to sepsis in aged mice, we collected colonic tissues from both aged and young septic mice for transcriptomic analysis. Differentially expressed genes were mainly enriched in pathways related to bacterial gene ontology (GO) functions, immune responses, response to bacterium and innate immune processes (Figure S5E). Nlrp6 is a key immune sensor in the gut that serves as a critical receptor for intestinal bacteria and their metabolites.^25^^,^^26^ Previous studies have suggested that Nlrp6 may also be a target of histamine produced by gut microbiota.^26^ Consistently, our results showed reduced Nlrp6 expression in aged septic mice compared to the young group (Figure S5F). Therefore, we hypothesized that HA contributes to sepsis, intestinal injury, and barrier dysfunction in aged mice by downregulating Nlrp6 expression and impairing intestinal autophagy.

**Figure 5.**
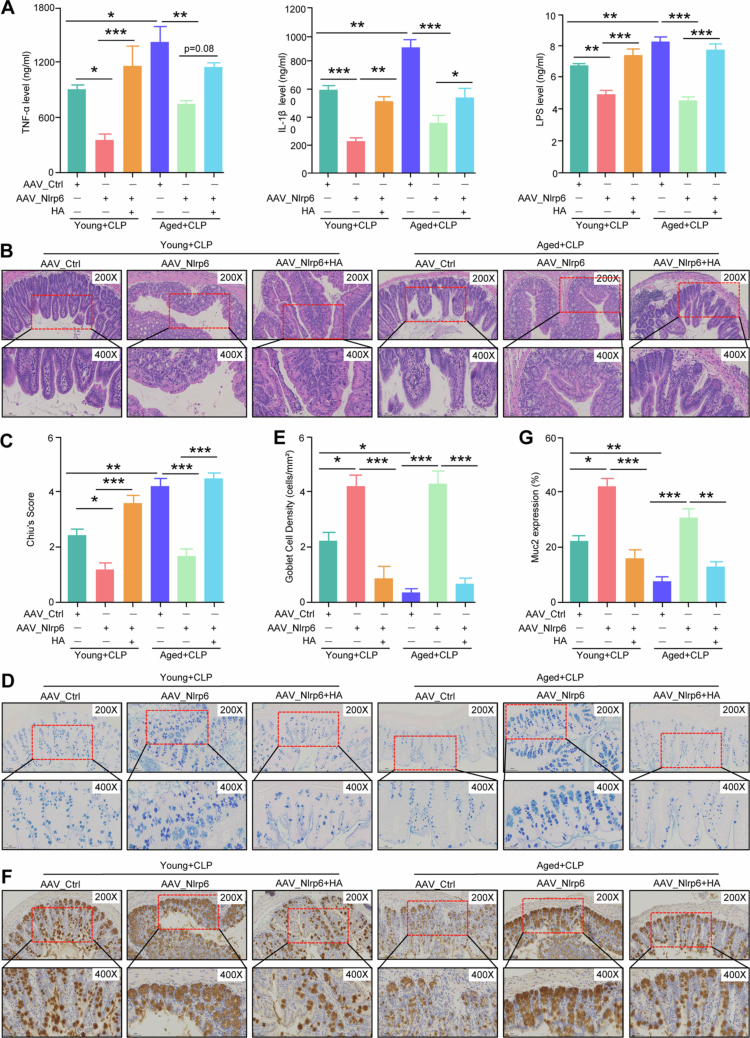
HA inhibits Nlrp6 expression to exacerbate intestinal barrier dysfunction in septic mice. (A) TNF-α, IL-1β and LPS in the serum from the young and aged septic mice treated with HA after pretreated with Nlrp6 overexpression. *n* = 4. *, *p* < 0.05; **, *p* < 0.01; ***, *p* < 0.001. (B,C) H&E staining (B) and histopathological score (C) of the colon from young and aged CLP mice subjected to pretreatment with HA after the Nlrp6 overexpression mice. *n* = 4. *, *p* < 0.05; **, *p* < 0.01; ***, *p* < 0.001. (D,E) AB‒PAS staining (D) and goblet cell number (E) of colon from young and aged CLP mice subjected to pretreatment with HA after the Nlrp6 overexpression mice. *n* = 4. *, *p* < 0.05; ***, *p* < 0.001. (F,G) Immunohistochemistry (F) and quantification of Muc2 expression (G) of colon from young and aged CLP mice subjected to pretreatment with HA after the Nlrp6 overexpression mice. *n* = 4. *, *p* < 0.05; **, *p* < 0.01; ***, *p* < 0.001.

To assess whether HA exacerbates intestinal permeability and systemic inflammation by inhibiting Nlrp6 expression in septic mice, we used ELISA to quantify serum concentrations of FITC, LPS, and cytokines. Systemic inflammatory responses were compared across different groups. Serum levels of FITC, LPS, TNF-α, and IL-1β were significantly higher in the aged group than in the young group. Notably, Nlrp6 overexpression reduced TNF-α, IL-1β, FITC, and LPS levels compared with the CLP group. However, HA intervention led to increased inflammatory response and greater intestinal barrier permeability compared to the adeno-associated virus-mediated Nlrp6 overexpression combined with CLP (AAV_Nlrp6+CLP) group (Figures 5A and S6A). These findings suggest that HA may promote inflammation and intestinal permeability in septic mice by downregulating Nlrp6 expression. These effects were especially pronounced in aged septic mice.

To further investigate the role of HA in mediating intestinal injury and barrier dysfunction through inhibition of Nlrp6 expression, we performed H&E and AB–PAS staining, immunohistochemical analysis, and WB assays. The findings indicated that aged septic mice exhibited more severe epithelial cell shedding in the upper and middle colon than young septic mice, along with pronounced villus shortening and widening and severe inflammatory infiltration, resulting in higher Chiu's scores. Nlrp6 overexpression markedly improved inflammatory infiltration, villus shortening, and epithelial shedding in septic mice, resulting in lower Chiu's scores compared with the CLP group. However, subsequent HA intervention significantly worsened these pathological changes, as indicated by shorter villi, increased ulcerative and inflammatory infiltration, and higher Chiu's scores compared with the AAV_Nlrp6+CLP group (Figures 5B−5C). These results indicate that HA may worsen intestinal injury by suppressing Nlrp6 expression in septic mice, with this effect being more pronounced in aged mice.

**Figure 6.**
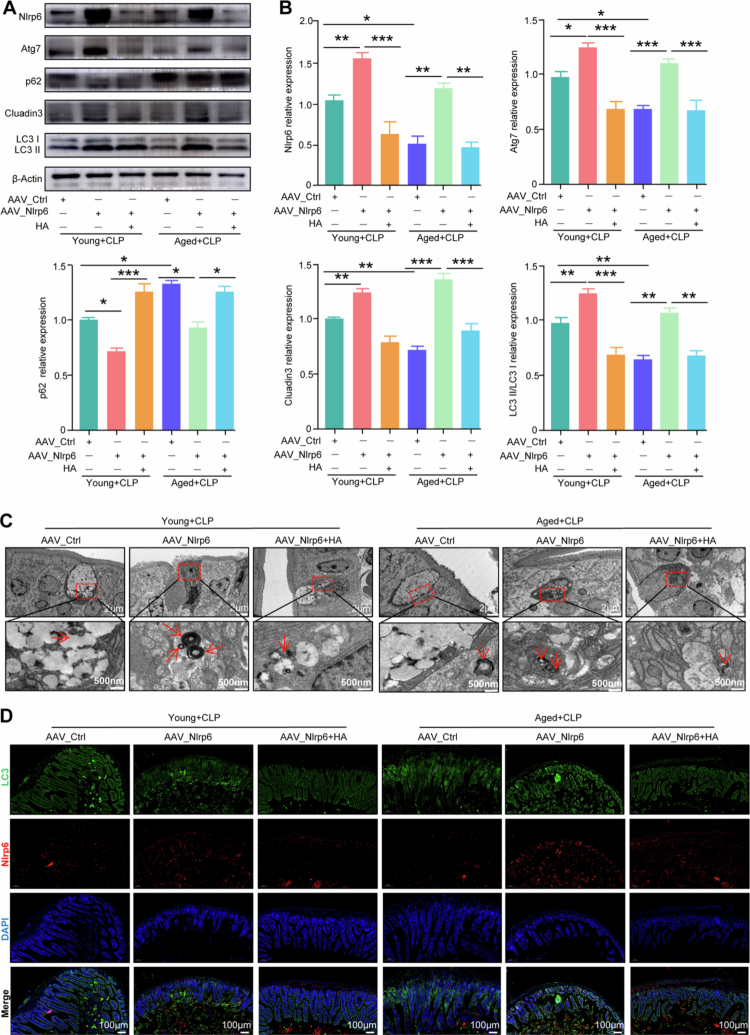
HA inhibits autophagy via regulating Nlrp6 expression to exacerbate sepsis-induced intestinal barrier dysfunction. (A) Western blot analysis for the quantification of protein expression of colon from young and aged CLP mice subjected to pretreatment with HA after the Nlrp6 overexpression mice. *n* = 5. (B) Semiquantitative analysis results of proteins of colon from young and aged CLP mice subjected to pretreatment with HA after the Nlrp6 overexpression mice. *n* = 5. *, *p* < 0.05; **, *p* < 0.01; ***, *p* < 0.001. (C) TEM for the quantification of autophagosome of colon from young and aged CLP mice subjected to pretreatment with HA after the Nlrp6 overexpression mice. *n* = 5. (D) Immunofluorescence analysis for the colocalization of Nlrp6 and LC3 in colon from young and aged CLP mice subjected to pretreatment with HA after the Nlrp6 overexpression mice. *n* = 4–5.

Additionally, AB-PAS staining and immunohistochemical analysis showed a decrease in goblet cell numbers and Muc2 expression in the colonic tissues of aged mice compared with the young group. Nlrp6 overexpression in septic mice further reduced goblet cell numbers and Muc2 expression compared with the CLP group. Notably, HA administration also reduced goblet cell numbers and Muc2 expression relative to the AAV_Nlrp6+CLP group (Figure 5D–G). Furthermore, the expression of the tight junction proteins Claudin1 and Claudin3 was higher in the AAV_Nlrp6+CLP group than in the CLP group. HA treatment reduced the expression of these proteins compared with the AAV_Nlrp6+CLP group (Figures 6A−6B and S6B−S6C). These findings demonstrate that HA impairs the intestinal mucus barrier by inhibiting Nlrp6 expression in septic mice, an effect that is more pronounced in aged mice. In summary, HA inhibits Nlrp6 expression, thereby promoting intestinal injury, barrier dysfunction, and inflammatory responses in septic mice, with these effects being amplified by aging.

### HA inhibits autophagy via regulating Nlrp6 expression to exacerbate sepsis-induced intestinal barrier dysfunction

To investigate whether HA influences autophagy in intestinal epithelial cells of septic mice by modulating Nlrp6 expression, we utilized WB assays, TEM, and confocal microscopy to assess autophagy-related proteins and autophagic flux. Additionally, we employed Caco2 cells to examine the induction of cellular senescence by H_2_O_2_. We confirmed successful senescence induction using β-galactosidase staining and flow cytometry to analyze the cell cycle (Figure S7A−S7B).

WB analysis of colon tissues revealed that Nlrp6, LC3, and Atg7 levels were reduced in the aged group. In the AAV_Nlrp6+CLP group, Nlrp6, LC3, and Atg7 expression was increased compared with the CLP group. However, HA intervention reduced the expression of Nlrp6, LC3, and Atg7 compared with the AAV_Nlrp6+CLP group (Figure 6A−6B). Protein expression in Caco2 cells mirrored that in the intestinal tissues of septic mice, confirming the reliability of both cellular and tissue models (Figure S7C−S7D). These findings indicate that HA exacerbates autophagy inhibition and increases intestinal barrier permeability by downregulating Nlrp6 expression.

TEM showed fewer autophagosomes in the intestinal tissues of the aged group compared with the young group. The number of autophagosomes increased in the AAV_Nlrp6+CLP group compared with the CLP group, while HA treatment reduced autophagosome formation compared with the AAV_Nlrp6+CLP group, indicating that HA impedes autophagosome formation by inhibiting Nlrp6 expression (Figure 6C). Similarly, confocal microscopy showed that autophagic flux in Caco2 cells from the aged group was lower than in those from young septic mice. Nlrp6 overexpression significantly enhanced autophagic flux compared with the LPS group, while HA treatment suppressed autophagic flux compared with the AAV_Nlrp6+CLP group (Figure S7). These results indicate that HA inhibits autophagic flux by suppressing Nlrp6 expression.

To elucidate the mechanism by which HA or aging suppresses autophagy via inhibition of Nlrp6 expression and its interaction with LC3, we performed colocalization fluorescence and coimmunoprecipitation (Co-IP) assays. Immunofluorescence analysis showed reduced colocalization of Nlrp6 and LC3 in colonic tissue from the aged group compared with the young group. In contrast, colocalization increased in the AAV_Nlrp6+CLP group compared with the CLP group, whereas HA treatment reduced co-localization compared with the AAV_Nlrp6+CLP group (Figure 6D). Similar results were observed in Caco2 cells, confirming consistency across experimental models (Figure S7F). Co-IP assays further confirmed that Aged may be associated with a reduction of the interacI on between Nlrp6 and LC3 compared with the young group (Figure S8). Collectively, these findings indicate that HA inhibits autophagy by suppressing Nlrp6 expression, thereby reducing the interaction between Nlrp6 and LC3 and contributing to autophagy deficiency in intestinal cells of septic mice. This effect is more pronounced in aged mice, highlighting increased vulnerability to HA-mediated autophagy suppression with aging (Graphical Abstract).

## Discussion

Intestinal barrier integrity is paramount in the pathogenesis of sepsis.^27^ Previous researches have highlighted that aging-induced intestinal injury and inflammation contribute to impaired intestinal barrier function,^28-30^ yet the underlying mechanisms remain poorly understood. The increased mortality observed in septic mice due to aging could be significantly linked to several factors: intestinal injury, autophagy deficiency in intestinal epithelial cells, intestinal barrier dysfunction, bacterial translocation from the intestines, and an exacerbated inflammatory response. Therefore, further investigation into how aging promotes autophagy deficiency and intestinal barrier dysfunction is essential to uncover new insights into the pathophysiology of sepsis in elderly populations. Notably, sepsis imposes a particularly severe impact on elderly patients, with 58%–65% of sepsis cases occurring in individuals aged 65 and above.^31^^,^^32^

Previous studies have indicated that inflammatory factors in the colon of aged septic mice are significantly increased compared to those in young septic mice.^15^ Aging contributes to a notable decline in host sensitivity, pathogen clearance, and detection efficiency, leading to prolonged immune response stimulation and heightened susceptibility to inflammation, thereby promoting the secretion of a multitude of proinflammatory factors.^33^^,^^34^ Our experimental results corroborate these findings, demonstrating more severe intestinal injury and inflammatory responses in aged septic mice compared to their younger counterparts. By administering CCs from aged and young mice intraperitoneally, we initially eliminated the possibility that differences in sepsis susceptibility were merely due to varying sources of gut infection associated with aging. Subsequent to ABX pretreatment, mortality rates between aged and young septic mice were comparable. However, FMT assays indicated that gut microbiota from aged donors increased the risk of death in septic mice. Interestingly, we observed a decreased abundance of *K. aero* in aged septic mice, suggesting that *Klebsiella* species, as opportunistic intestinal pathogens, may enhance susceptibility to sepsis in older individuals.^35^ Nevertheless, the specific impact of *K. aero* on the progression of sepsis remains to be fully understood. These findings suggest that intragastric administration of *K. aero* could exacerbate sepsis by intensifying the inflammatory response. Collectively, our data underscore that restoring fecal microbiota homeostasis represents a promising strategy for sepsis intervention.

Metabonomic analysis in our study indicated that *K. aero* drives HA production, a critical intestinal-derived metabolite implicated in sepsis regulation.^36^ A previous study highlighted the capability of human gut microbiota, including *Bacteroides ovatus*, to liberate *N*-methylserotonin from orange fibers, showcasing the complex interactions within the gut ecosystem.^37^ In our research, we observed a notable increase in the proportion of *Klebsiella* at the genus level and specifically *K. aero* at the species level in aged septic mice. This increase was associated with more severe intestinal injury, barrier dysfunction, and inflammation, aligning with findings from previous studies.^11^^,^^38^

Past studies have highlighted that active intervention with *K. aero* in septic animals not only impacts myocardial function and ultrastructure but is also linked to variations in high-energy phosphate levels after 24 h.^39^ *K. aero* is additionally associated with nosocomial infections, such as hospital-acquired and ventilator-associated pneumonia.^40^ It has been demonstrated to induce sepsis, particularly in models of antibiotic resistance *K. aero.*^41^ HA, a biologically active monoamine, is synthesized by the enzymatic action of *HDC* in various cells. Recent studies suggest that under pathogenic stimulation, certain myeloid cells can also produce HA via *HDC.*^42^

Furthermore, it has been shown that strains of *K. aero* and *Klebsiella pneumoniae* from fish intestinal bacteria possess decarboxylase activity capable of converting amino acids into biogenic amines, including HA.^43^ Intriguingly, research indicates that intestinal strains of *K. aero*, rather than *K. pneumoniae* or *Klebsiella acidophilus*, are predominant producers of HA.^44^

In our study, we discovered that the genome of *K. aero* contains *HDC* genes, which facilitate the release of HA. Interestingly, *HDC*-deficient mutant strains of *K. aero* released higher concentrations of HA in culture supernatants than the wild-type strain. Furthermore, deletion of the *HDC* gene in *K. aero* ameliorated its adverse effects on intestinal barrier dysfunction in septic mice. It is noteworthy that *K. aero*, as a producer of HA, is influenced by the concentration of sodium chloride in its environment.^45^ Additionally, the production of HA by fish intestinal bacteria *K. aero* is affected by both the *HDC* gene and the pH value within the bacteria.^46^

A previous study suggested that HA could serve as a biomarker for predicting the prognosis of sepsis, potentially allowing for the identification of phenotypic associations at the early stages of the condition.^47^ Elevated HA levels have been correlated with the onset and worsening outcomes of sepsis.^48^ Recent research indicates that an increase in HA levels is a significant metabolic change in septic rats, a phenomenon that is more pronounced with aging,^49^ aligning with our findings. Collectively, these observations underscore the complex interplay between intestinal microbiota imbalance and impaired intestinal barrier function in aged septic hosts.

Research using CLP or LPS-induced sepsis animal models has demonstrated that the expression of *HDC* genes and the levels of HA in various organs correlate with increased *HDC* gene expression and HA levels.^50^ Studies utilizing HDC gene-deficient mice have revealed that the absence of HA leads to reduced levels of proinflammatory cytokines such as TNF-α, IL-1β, and IL-6, suggesting that HA can promote the production of proinflammatory factors.^50^ Conversely, another study reported that serum levels of proinflammatory cytokines were elevated in HDC gene-deficient mice, indicating potential anti-inflammatory activities of HA.^51^

Further studies have found that HA inhibits the expression of the Nlrp6 inflammasome, leading to impaired autophagy and exacerbating the inflammatory response and immune dysregulation in the host.^20^^,^^26^ Our findings support this, showing that HA can suppress Nlrp6 expression, thereby reducing the interaction between Nlrp6 and LC3 and inhibiting autophagy in intestinal epithelial cells. This suppression aggravates the inflammatory cascade, intensifying sepsis-related intestinal injury and barrier dysfunction, effects that are more pronounced in aging. Additionally, we observed decreased LC3 expression and increased p62 expression, further indicating autophagy deficiency during sepsis.

Utilizing a double-labeled adenovirus to monitor autophagy flow, we observed a decrease in autophagy levels during sepsis. Subsequent overexpression of the Nlrp6 gene significantly ameliorated the observed autophagy deficiency and improved autophagy flux. However, HA intervention negated the beneficial effects of Nlrp6 overexpression, blocking the enhancement of autophagy flux. This is in line with a recent study which noted an increase in the ratio of LC3II to LC3I and elevated p62 expression, indicative of obstructed autophagy flux in sepsis.^52^ During natural aging, decreased LC3 expression and increased p62 indicate that aging intrinsically suppresses autophagy.^53^ More importantly, previous studies have reported a significant age-dependent decline in autophagy in endotoxin-induced myocardial toxicity, with higher mortality associated with autophagy deficiency,^54^ consistent with our findings. Furthermore, the regulatory effect of HA on autophagy is context dependent and varies across different diseases. For example, histamine promotes neutrophil adhesion by inhibiting autophagy in dairy cows with subacute ruminal acidosis,^55^ whereas histamine H1R antagonist treatment attenuates subarachnoid hemorrhage pathology by activating Nrf2/SQSTM1-mediated autophagy.^56^ Conversely, histamine deficiency aggravates cardiac injury by modulating myocardial autophagy and apoptosis under hypoxic and acute myocardial infarction conditions.^57^ In our study, elevated HA levels contributed to autophagy deficiency and increased susceptibility to sepsis. Inhibition of Nlrp6 expression led to autophagy deficiency and further exacerbated intestinal barrier dysfunction in septic mice.

Autophagy is crucial for maintaining intestinal homeostasis. Previous research has demonstrated that autophagy deficiency in intestinal epithelial cells can lead to bacterial translocation across the intestinal barrier and trigger inflammatory cascades in extraintestinal organs.^58^ Nlrp6 is known to stabilize the intestinal microenvironment.^25^ Our data indicate that *K. aero* containing *HDC* can convert histidine into HA. HA not only drives intestinal barrier dysfunction by inhibiting Nlrp6 expression—thereby reducing the interaction between Nlrp6 and LC3—but also exacerbates autophagy deficiency in intestinal epithelial cells. This leads to decreased Muc2 secretion, promoting further intestinal barrier dysfunction and bacterial translocation in septic mice, effects that are exacerbated during aging.

In summary, our findings enhance understanding of how fecal microbiota dysbiosis in aging contributes to intestinal barrier dysfunction. Specifically, this dysfunction occurs through the inhibition of Nlrp6 expression, which subsequently reduces the interactions between Nlrp6 and LC3, exacerbating autophagy deficiency. This deficiency impairs Muc2 secretion, thereby increasing the host's susceptibility to intestinal injury induced by sepsis.

### Study limitations

Despite our findings that clearly demonstrate the mediating role of Nlrp6 in linking intestinal flora imbalance to barrier dysfunction in aged sepsis hosts, the mechanisms by which HA inhibits Nlrp6 expression in this context remain incompletely clarified. Additionally, while this study focused on the colon due to its high Muc2 and Nlrp6 expression and central role in mucus barrier maintenance, sepsis-induced damage in the jejunum and ileum warrants further investigation. Future studies will systematically compare regional responses to delineate segment-specific mechanisms. Moreover, our study was specifically focused on intestinal barrier dysfunction in aging sepsis hosts, without a stratified age analysis, which limits the generalizability of our results across different age groups. The relationship between intestinal flora imbalance and infectious diseases across these groups has not been clearly defined. Finally, the underlying reasons for the observed increase in *K. aero* prevalence in aging also warrant further investigation to fully understand their implications and mechanisms.

## Methods

### Experimental model and subject data

#### Animals

We utilized specific pathogen-free (SPF) C57BL/6 mice, grouped by age into young (8–10 weeks) and aged (22–24 months) groups. All animals were housed at a controlled temperature of 22–24 °C with ad libitum access to food and water under a 12 h light–dark cycle. The animal experimental procedures were conducted in accordance with the National Institutes of Health guidelines and received approval from the Committee of Zhengzhou University, Henan, China. Importantly, the study protocols were approved by the Institutional Ethical Committee, with approval numbers 2022-KY-0215 and 2024-KY-1244.

### CLP-induced septic mouse model

The CLP-induced sepsis mouse model was generated following previously described protocols.^59^ The mice were deeply anesthetized, and a 1–2 cm midline laparotomy was performed under sterile conditions. Half of the distal cecum was ligated and punctured once with a 21 G needle, allowing a small amount of fecal content to be extruded from the perforation site. The incision was then sutured layer by layer, and 1 ml of sterile normal saline was administered subcutaneously for resuscitation.

To explore the effects of HA on septic mice, 20 mg/Kg of HA (MedChemExpress, Monmouth Junction, NJ, USA) dissolved in sterile water was administered intraperitoneally before CLP. Additionally, the mice received intravenous treatment with adeno-associated-virus-9 (AAV9) targeting Nlrp6 (Hanbio Tech, Shanghai, China) at a dose of 1.4 × 10^11^ Vg per mouse 3‒4 weeks prior to CLP to induce overexpression of the Nlrp6 gene. Furthermore, HA was administered intraperitoneally to AAV9-Nlrp6 mice 2 h before CLP. All the samples were collected from all mice 8 h after either sham operation or cecal ligation and puncture. The mice were euthanized according to the specific requirements of the study.

### Septic peritonitis model induced by CCs

CCs were harvested from both young and aged mice and then suspended at a 1:10 dilution with phosphate-buffered saline (PBS) based on weight/volume. Subsequently, 500 µl of the CC suspension was injected intraperitoneally into young mice to induce septic peritonitis.

### Septic patients

Fresh fecal samples were collected from both young and aged septic patients and were immediately frozen at −80 °C for preservation. The baseline characteristics of the septic patients are presented in Table S1. These fecal samples were obtained in accordance with the Sepsis 3.0 criteria from the First Affiliated Hospital of Zhengzhou University. The study protocols were approved by the Institutional Ethical Committee, with approval numbers 2021-KY-0467-003 and 2022-KY-0215.

### Cell culture

Caco2 cells (h032, iCell) were cultured following the manufacturer's instructions. To induce senescence, the cells were treated with 200 μmol/L of 30% hydrogen peroxide (H_2_O_2_, Sigma-Aldrich (Shanghai) Trading Co., Ltd.) for 2 h, followed by a recovery period of 72 h (termed the aged group). For the overexpression of the Nlrp6 gene, Caco2 cells were seeded at a density of 4 × 10^5^ cells per ml using the pcDNA3.1 Nlrp6 vector (Genechem, Shanghai, China). The vector was constructed with the forward primer 5′-CACACTGGACTAGTGGATCCCGCCACCATGGACCAGCCAGAGGCCCCCTGCTCC-3′ and the reverse primer 5′-AGTCACTTAAGCTTGGTACGAAGGTCGAGATGAGTTCCTTGGGAGG-3′. Transfection was carried out using Lipofectamine™ 3000 (ThermoFisher, MA, USA) according to the manufacturer's guidelines, with protein expression analyzed 36 h post-transfection.

Additionally, the mRFP-GFP-LC3 vector (Hanbio Tech, Shanghai, China) was transfected into Caco2 cells as per the manufacturer's instructions. Autophagic flux was assessed using confocal microscopy 36 h after transfection.

### Bacterial culture

*K. aero* was cultured in Luria-Bertani (LB) medium at 37 °C with shaking at 180 rpm for 10 h under aerobic conditions. For the animal experiments, mice received intragastric administration of *K. aero* at a dose of 2 × 10^8^ CFU per mouse once daily for 3 d. Subsequently, the mice were subjected to CLP treatment.

## Method information

### Gut microbiota depletion and transplantation

To deplete the intestinal microbiota, mice were treated orally with a broad-spectrum antibiotic cocktail consisting of 100 mg/Kg vancomycin, 200 mg/Kg neomycin sulfate, 200 mg/Kg metronidazole, and 200 mg/Kg ampicillin for 3 d.^16^ Following this treatment, approximately 3 g of fecal material from either aged or young septic mice was resuspended in PBS to achieve a fecal concentration of 0.125 g/ml. This suspension was then administered intragastrically to young recipient mice. These recipient mice were subsequently utilized for further experimental assessments.

### Microbial analysis

Total genomic DNA was extracted from cecal feces stored at −80 °C using the cetyltrimethylammonium bromide/sodium dodecyl sulfate (CTAB/SDS) method. The extracted DNA was diluted to a concentration of 1 ng/μl with sterile water for subsequent analyzes. Amplification of the 16S rDNA V3-V4 regions was performed using specific primers (341F: CCTAYGGGRBGCASCAG; 806 R: GGACTACNNGGGTATCTAAT) that included a barcode for identification. The PCR products were sequenced on the Illumina NovaSeq 6000 platform.

Furthermore, we also conducted metagenomic sequencing, with the specific steps as follows: the total DNA was extracted from samples using the Fecal Genome DNA Extraction Kit (AU46111-96, BioTeke, China). DNA libraries were constructed using the TruSeq Nano DNA Library Preparation Kit-Set (#FC-121-4001, Illumina, USA) following the manufacturer's instructions. Metagenome libraries were then sequenced on an Illumina NovaSeq 6000 platform with PE150. Fastp software (v0.23.4) were used to remove the reads that contained adapter contamination, low-quality bases and undetermined bases. The sequence quality was subsequently verified using fastp. The quality-filtered reads were first aligned to the genome by using bowtie (v2.2) to filter out host contaminations. Then, the remaining reads were subjected to de novo assembly for each sample using MEGAHIT (v1.2.9) and used to assign microbial functions and taxonomy. Metaprodiga(v2.6.3) was used to predict the coding regions (CDS) of the assembled contigs, and the CDS sequences of all the samples were clustered using MMseq2 (v15-6f452) to obtain unigenes. DIAMOND (v 0.9.14) was used to perform a taxonomic assessment of the microbiota based on the NR database. The Wilcoxon test was used to identify the differentially abundant species, and significances were declared at *p* < 0.05 and |log2-fold change|>1.

### Construction of the deficient mutant of *K.aero* strain

We constructed the *HDC*-deficient mutant of *K. aero* using double homologous recombination. Initially, single clones were inoculated in liquid medium with corresponding antibiotic resistance and cultured overnight at 37 °C to confirm antibiotic resistance. Genomic DNA was extracted from these cultures using the PureLink™ Microbiome DNA Purification Kit (A29790, Thermo). This DNA served as a template to amplify the *HDC* knockout regions using the upper and lower homology arms. The primer sequences used were: HDC-19-F/HDC-up-R: TGTAAAACGACGGCCAGTTATTTTCCAGTACCGCACGCATA/ACATAAATATGGAGATTCATGAGTGAGCAGCGGCAAACTG;HDC-down-F/HDC-19-R:GTTTGCCGCTGCTCACTCATGAATCTCCATATTTATGTTG/CTATGACCATGATTACGCCATGTCCCGATGAAAGGTCTGATT.

The gel-purified PCR fragments of the *HDC* gene were used for subsequent ligation. The purified *HDC* UP and *HDC* DOWN fragments were ligated into the pUC19 vector backbone. This construct was transformed into E. coli DH5α competent cells, and colonies were selected on Amp-IPTG-X-Gal plates using the blue‒white screening method. Positive clones were confirmed by Sanger sequencing to determine whether the fragment had the intended mutations.

The extracted plasmids from the verified positive clones served as templates for amplifying the *HDC* repair homology arm using primers *HDC*-up-F and *HDC*-down-R. The PCR products were then digested and purified, ready for CRISPR/Cas9 gene editing.

For transformation, 10 µl of plasmid containing Cas9 was added to the prepared competent cells and kept on ice for 5 min. The mixture was then electrotransformed at 2500 V, followed by the addition of 1 ml of LB medium and incubation at 37 °C for 1 h. Transformed cells were plated on kanamycin-resistant plates to select positive clones.

Cas9-expressing competent cells were prepared similarly and used for a subsequent transformation with the hdc repair homology arm and the prepared hdc sgRNA plasmid. After transformation, cells were plated on kanamycin/tetracycline plates to select for *HDC* deletion strains. Positive *HDC*-deficient clones were identified using primers *HDC*-up-F (TATTTTCCAGTACCGCACGCATA) and *HDC*-down-R (ATGTCCCGATGAAAGGTCTGATT) and further verified by Sanger sequencing.

### ELISA assays

The concentrations of TNF-α, IL-1β, LPS, and HA were quantified using ELISA kits provided by Cusabio and Elabscience, both located in Wuhan, China. All assays were performed strictly according to the manufacturer's instructions.

### qRT‒PCR experiment

Total RNA was extracted using TRIzol reagent (Solarbio, Beijing, China). Reverse transcription was performed using a TaqMan Reverse Transcription Kit (UE, Suzhou, China). Quantitative PCR was conducted using qPCR SuperMix (Yeasen, Shanghai, China), and the results were analyzed using the 2^−ΔΔCT^ method to quantify gene expression levels. Details of all the qRT‒PCR primers used are provided in Table S2.

### Immunoprecipitation

Caco2 cells were cultured at a density of 4 × 10^5^ cells per ml. The pcDNA3.1 Nlrp6 plasmid with a Flag tag was used to achieve targeted overexpression of Nlrp6 in Caco2 cells. Transfection was carried out using Lipofectamine™ 3000 (ThermoFisher, MA, USA) following the manufacturer's instructions, with the transfection process continuing for 12 h. Subsequently, transfection with mRFP-GFP-LC3 (Hanbio Tech, Shanghai, China) was performed according to the manufacturer's guidelines, and 36 h post-transfection, the cells were incubated overnight at 4 °C with an anti-Nlrp6 antibody (Bioss, Beijing, China). The cells were then incubated with Dynabeads™ Protein G magnetic beads (Invitrogen, CA, USA) at 4 °C to facilitate the immunoprecipitation. WB analysis was subsequently performed using Nlrp6 antibodies (Bioss, Beijing, China) and LC3 antibodies (CST, MA, USA) to detect the proteins of interest.

### Western blot

Caco2 cells and colon tissues were lysed using RIPA buffer (Solarbio, Beijing, China) on ice, followed by denaturation in SDS loading buffer by heating for 10 min. WB was conducted according to the manufacturer's guidelines. The proteins were probed using the following primary antibodies: β-actin (Abcam, MA, USA), LC3 (CST, MA, USA), Nlrp6 (Bioss, Beijing, China), Atg7 (CST, MA, USA), p62 (CST, MA, USA), Occludin (Proteintech, Wuhan, China), Claudin1 (Abcam, MA, USA), and Claudin3 (Abcam, MA, USA). Secondary antibodies were sourced from Proteintech (Wuhan, China). Protein expression was detected using an enhanced chemiluminescence (ECL) assay (UE, Suzhou, China).

### Histopathological analysis

Colon tissues from septic mice were collected and fixed in 4% paraformaldehyde for 24 h. The tissues were then embedded in paraffin, and histological sections were prepared for H&E staining. The severity of intestinal injury was evaluated based on parameters such as congestion, intestinal wall thickening, ulceration, and adhesion. These features were scored using a standardized scale ranging from 0 (absent) to 4 (severe), as previously described.^60^ The stained sections were examined under a microscope (NanoZoomer S60, Hamamatsu, Japan).

### Flow cytometry

To assess apoptosis and evaluate cellular senescence, we utilized the FITC Annexin V Apoptosis Detection Kit I (BioLegend, CA, USA). Caco2 cells were first washed twice with cold PBS and then resuspended in 1× binding buffer to a concentration of 1 × 10^6^ cells/ml. Next, 100 µl of this cell suspension (1 × 10^5^ cells) was transferred to a 5 ml culture tube. To each tube, 5 µl of FITC Annexin V and 5 µl of propidium iodide (PI) were added. After gently vortexing, the cells were incubated for 15 min at room temperature in the dark. Following incubation, 400 µl of 1× binding buffer was added to each tube. Flow cytometric analysis was conducted within 1 h to ensure optimal assessment of the cells.

### Immunohistochemical assays

Paraffin-embedded tissue sections were dewaxed and rehydrated before being incubated with 3% hydrogen peroxide for 20 min to block endogenous peroxidase activity. The sections were then blocked with goat serum for 30 min to prevent nonspecific binding. Subsequently, the sections were incubated overnight at 4 °C with an anti-Muc2 antibody (Servibio, Wuhan, China). Following this, sections were incubated with a biotinylated secondary antibody and streptavidin-coupled horseradish peroxidase (HRP). After three washes, the sections were stained with diaminobenzidine (DAB) and counterstained with hematoxylin. For each sample, at least three fields were randomly selected for observation. Images were captured and analyzed using ImageJ software.

### Fluorescence staining

Immunofluorescence colocalization of Nlrp6 and LC3 was performed in both Caco2 cells and colon paraffin-embedded tissue sections following the manufacturer's instructions. Initially, both cells and tissue sections were incubated overnight at 4 °C with a primary antibody against Nlrp6 (Bioss, Beijing, China). For detection, tissue sections were incubated with Cy3-conjugated donkey anti-rabbit IgG (Servicebio, Wuhan, China), and cells with iF488-Tyramide (Servicebio, Wuhan, China) for 1 h at room temperature. Afterward, a 10min incubation with Tyramide signal amplification (TSA) was conducted in the dark, with the tissue sections also undergoing antigen retrieval using an antigen repair buffer. Subsequently, cells and tissue sections were incubated overnight at 4 °C with an antibody against LC3 (CST, MA, USA). For visualization, cells were treated with Cy3-conjugated donkey anti-rabbit IgG (Servicebio, Wuhan, China), and tissue sections with iF488-Tyramide (Servicebio, Wuhan, China), each for 1 h at room temperature. Finally, DAPI (Servicebio, Wuhan, China) was applied to stain the nuclei.

### Alcian blue PAS (AB‒PAS) staining

Paraffin-embedded tissue sections were stained using the Alcian Blue PAS staining kit (Solarbio, Beijing, China) to evaluate the number of goblet cells. Stained sections were then examined under a microscope (NanoZoomer S60, Hamamatsu, Japan) for detailed analysis.

### Transmission electron microscopy (TEM)

Fresh tissue blocks were collected promptly within 1–3 min specifically for TEM analysis. The tissues were fixed overnight at 4 °C using fresh TEM fixative, composed of 2.5% glutaraldehyde in PBS. Following initial fixation, tissues were further fixed with 1% osmium tetroxide in PBS at room temperature for 2 h. The samples were then dehydrated in a graded series of ethanol, infiltrated, and embedded in resin. They were subsequently polymerized in a 65 °C oven. Ultrathin sections (60–80 nm) were cut and placed on copper grids for examination. Micrographs were obtained using a Jeol JEM 1400 Plus electron microscope (Jeol, USA).

### Intestinal permeability assays

Septic mice were fasted for 8 h prior to the assay. FITC-dextran (3–5 kDa, Sigma, MA, USA) was then administered intragastrically at a dose of 60 mg/Kg. Blood samples were collected 3 h postadministration. The fluorescence intensity of the samples was measured using a microplate reader equipped with a multiwavelength measurement system, setting the excitation at 488 nm and emission at 520 nm.

### Metabolomics analysis

Approximately 25 mg of each sample was weighed and placed into an EP tube, to which 500 μl of extraction solution was added. The extraction solution consisted of methanol, acetonitrile, and water in a 2:2:1 ratio, enriched with an isotopically-labeled internal standard mixture. The mixture was thoroughly vortexed and centrifuged, and the resulting supernatant was transferred to a fresh glass vial for subsequent analysis. LC–MS/MS analyzes were conducted using a Vanquish UHPLC system (Thermo Fisher Scientific) equipped with a UPLC BEH amide column (2.1 × 100 mm, 1.7 μm). The system was coupled to a Q Exactive HFX mass spectrometer (Orbitrap MS, Thermo). Metabolites were annotated using an in-house MS2 database (BiotreeDB) to facilitate accurate metabolite identification.

Sample preparation: Samples were initially centrifuged at 12,000 rpm for 15 min at 4 °C. After centrifugation, 20 µl of H_2_O was added to 40 µl of the supernatant. This mixture was homogenized and sonicated in three cycles, then left to settle at −20 °C overnight. Following this, the samples were again centrifuged at 12,000 rpm and 4 °C for 15 min.

Chemical derivatization: 80 µl of the resulting supernatant was transferred into an EP tube. The samples underwent derivatization by adding 40 µl of 100 mmol/L sodium carbonate solution and 40 µl of 2% benzoyl chloride in acetonitrile, followed by incubation for 30 min. After derivatization, 10 µl of an internal standard was added, and the samples were centrifuged at 12,000  rpm for 15 min at 4 °C.

UHPLC-MS/MS analysis: 40 µl of the supernatant was diluted with 20 µl H_2_O and transferred to an auto-sampler vial. The UHPLC separation was performed using an ACQUITY Premier (Waters) system equipped with a Waters ACQUITY UPLC HSS T3 column (100 × 2.1 mm, 1.8 µm). The mobile phase consisted of 0.1% formic acid and 1 mM/L ammonium acetate in water (Phase A) and acetonitrile (Phase B). The column temperature was maintained at 40 °C, with the autosampler temperature set at 10 °C. The injection volume was 1 µl. Detection was carried out using a SCIEX Triple Quad™ 6500+ mass spectrometer.

### Transcriptomic analysis

Colonic tissues were collected for RNA extraction using TRIzol reagent. Subsequent library construction and Illumina sequencing were performed to evaluate gene expression. These processes were conducted by Novogene Co., Ltd. (Beijing, China).

## Quantification and statistical analysis

Data are presented as the mean ± standard error of the mean (SEM). Statistical analyzes were performed using one-way analysis of variance (ANOVA) followed by Tukey's multiple comparison test, and unpaired t tests where appropriate. A *p*-value of less than 0.05 was considered statistically significant. Graphical representations in the abstract were generated using BioRender.

## Supplementary Material

Table S2.docx Table S2.docx

Table S1.docx Table S1.docx

Supplementary material Supplementary figures

## Data Availability

All data supporting the findings of this study are available within the article or from the corresponding author upon reasonable request. The 16S rRNA gene sequencing raw data have been deposited in the NCBI database (accession numbers: PRJNA1394304 and PRJNA1394310).
