# Supplementary material for: Aging-caused the changes of the gut microbiota drive intestinal barrier dysfunction and increase sepsis susceptibility
Source: Gut Microbes. 2026 Feb 21;18(1):2630475. doi: 10.1080/19490976.2026.2630475 (PMC12928652; doi:10.1080/19490976.2026.2630475)
Supplement: Table S2.docx [file KGMI_A_2630475_SM5043.docx]

|  | **Forward primer (5**′**-3**′**)** | **Reverse primer (5**′**-3**′**)** |
| --- | --- | --- |
| *TNF-α* | CAGGCGGTGCCTATGTCTC | CGATCACCCCGAAGTTCAGTAG |
| *Ccl2* | TAAAAACCTGGATCGGAACCAAA | GCATTAGCTTCAGATTTACGGGT |
| *Cxcl1* | AATGAGCTGCGCTGTCAGT | ACTTGGGGACACCTTTTA |
| *β-Actin* | ATGGAGGGGAATACAGCCC | TTCTTTGCAGCTCCTTCGTT |
| *K.aero* | GTAACCGGTGAAACCGAAAGC | GATGCCGCCTTCGTAGTGGAAATGG |
| *16S* | ACTCCTACGGGAGGCAGCAG | ATTACCGCGGCTGCTGG |

**Table S2. Primers for qRT-PCR.**
