# Supplementary material for: Aging-caused the changes of the gut microbiota drive intestinal barrier dysfunction and increase sepsis susceptibility
Source: Gut Microbes. 2026 Feb 21;18(1):2630475. doi: 10.1080/19490976.2026.2630475 (PMC12928652; doi:10.1080/19490976.2026.2630475)
Supplement: Table S1.docx [file KGMI_A_2630475_SM5042.docx]

**Table S1. Baseline characteristics of septic patients.**

| **Parameters** | **Young** | **Aged** |
| --- | --- | --- |
| **No. of cases (%)** | 27(52.9%) | 24(44.1%) |
| **Age (Years)** | 52.8±8.9 | 74.6±7.8 |
| Median (range) | 57(36-63) | 74(66-94) |
| **Gender** |  |  |
| Male (%) | 15(60.0%) | 14(70.0%) |
| Female (%) | 10(40.0%) | 6(30.0%) |
| **SOFA score (mean±SD)** | 12.3±5.8 | 13.7±5.5 |
| **Apache II score (mean±SD)** | 15.8±6.6 | 12.8±5.7 |
| **In-hospital mortality (%)** | 0(0.0%) | 2(10.0%) |

**Note:**Among them, 12 aged patients and 13 young septic patients were used for Figure 2, Figure 3 and Figure S4; the feces from another 12 aged patients and 14 young sepsis patients were used to verify the fecal transplantation efficiency of the FMT experiment in Figure S3.
