# Supplementary material for: Aging-caused the changes of the gut microbiota drive intestinal barrier dysfunction and increase sepsis susceptibility
Source: Gut Microbes. 2026 Feb 21;18(1):2630475. doi: 10.1080/19490976.2026.2630475 (PMC12928652; doi:10.1080/19490976.2026.2630475)
Supplement: Supplementary material — Supplementary figures [file KGMI_A_2630475_SM5220.docx]

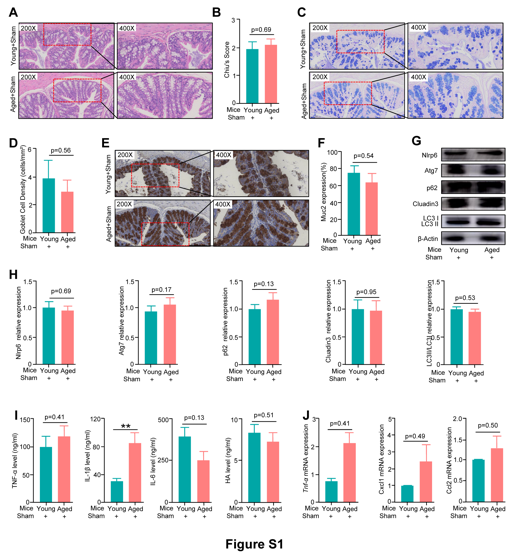


**Figure S1. The aged Sham-treated mice have similar phenotype to the young sham-operated mice.**

(A-B) H&E assay (A) and histopathological score (B) in the colon from young and aged Sham-treated mice. n = 5. (C-D) AB-PAS staining (C) and goblet cell density (D) of colon from young and aged Sham-treated mice. n = 4-5. (E-F) Immunohistochemistry (E) and quantification of Muc2 expression (F) of colon from young and aged Sham-treated mice. n =5.(G-H)Western blot analysis for the quantification of protein expression of colon from young and aged Sham-treated mice. n =5.(I)Serum TNF-α, IL-1β, IL-6 and HA levels from young and aged Sham-treated mice. n = 4-5.(J)Quantification of cytokines mRNA expression in the colon from young and aged Sham-treated mice. n = 4-5.


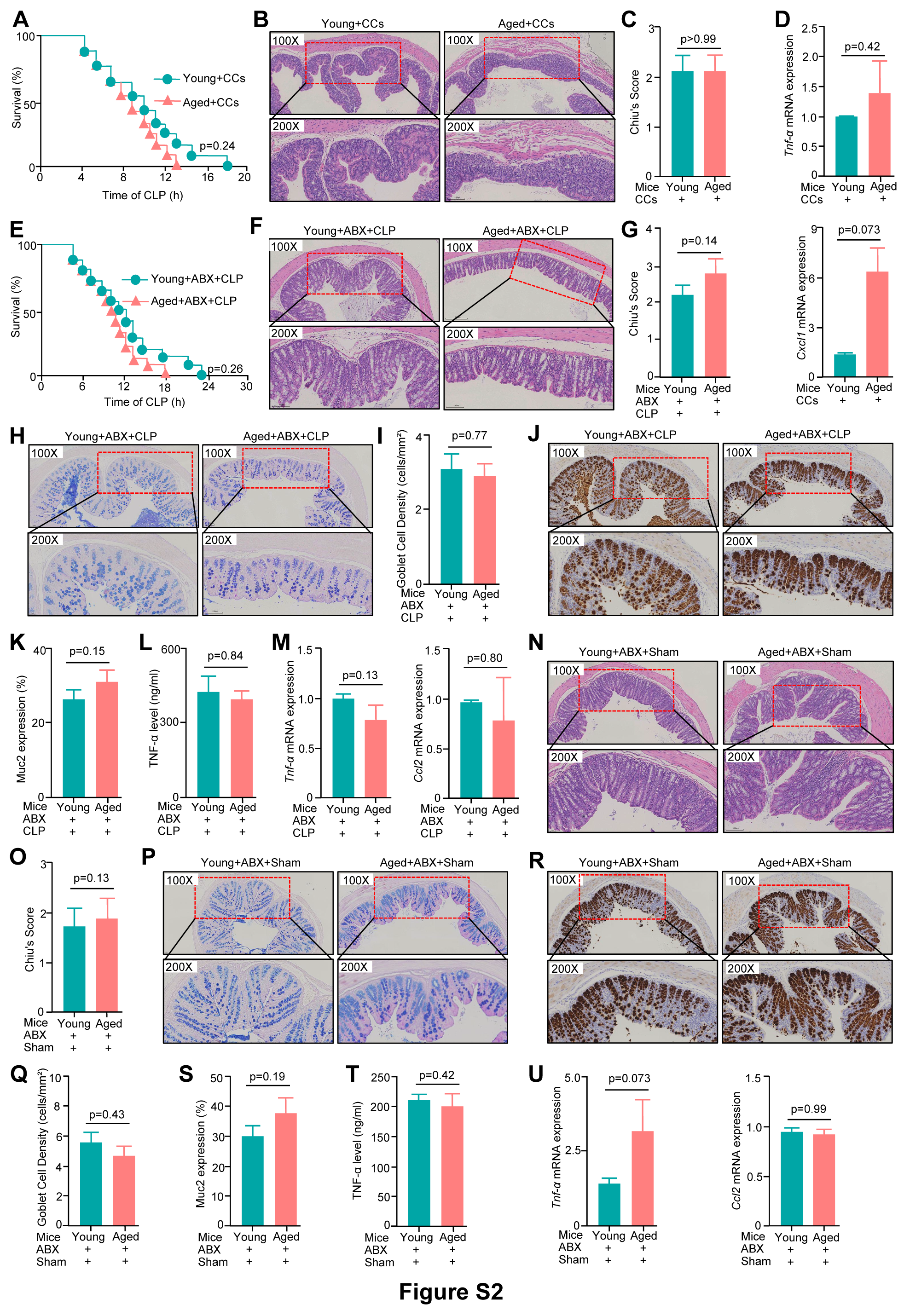


**Figure S2. The impact of gut microbiota changes to sepsis susceptibility in aged mice through cecal content injection**

(A) The survival rate in young septic mice resulted in intraperitoneal injections of CCs from the young and aged mice. n = 10. (B-C) H&E staining (B) and histopathological score (C) in the colon of young septic mice resulted in intraperitoneal injections of CCs from the young and aged mice. n = 5.(D) Quantification of cytokines mRNA expression in the colon of young septic mice resulted in intraperitoneal injections of CCs from young and aged mice. n =5.(E) The survival rate in ABX-pretreated young and aged mice with CLP treatment. n = 12.(F-G) H&E staining (F) and histopathological score (G) in the colon of ABX-pretreated young and aged mice with CLP treatment. n = 5.(H-I) AB-PAS staining (H) and goblet cell density (I) in the colon of ABX-pretreated young and aged mice with CLP treatment. n = 5. (J-K)Immunohistochemistry (J) and quantification of Muc2 expression (K) in the colon of ABX-pretreated young and aged mice with CLP treatment. n =5.(L-M) Quantification of cytokines in the serum and colon ABX-pretreated young and aged mice with CLP treatment. n = 5-6.(N-O) H&E staining (N) and histopathological score (O) in the colon of ABX-pretreated young and aged mice with sham treatment. n = 5.(P-Q) AB-PAS staining (P) and goblet cell density (Q) in the colon of ABX-pretreated young and aged mice with sham treatment. n = 4-5. (R-S)Immunohistochemistry (R) and quantification of Muc2 expression (S) in the colon of ABX-pretreated young and aged mice with sham treatment. n =5.(T-U) Quantification of cytokines in the serum(T) and colon(U) ABX-pretreated young and aged mice with sham treatment. n = 4-6.


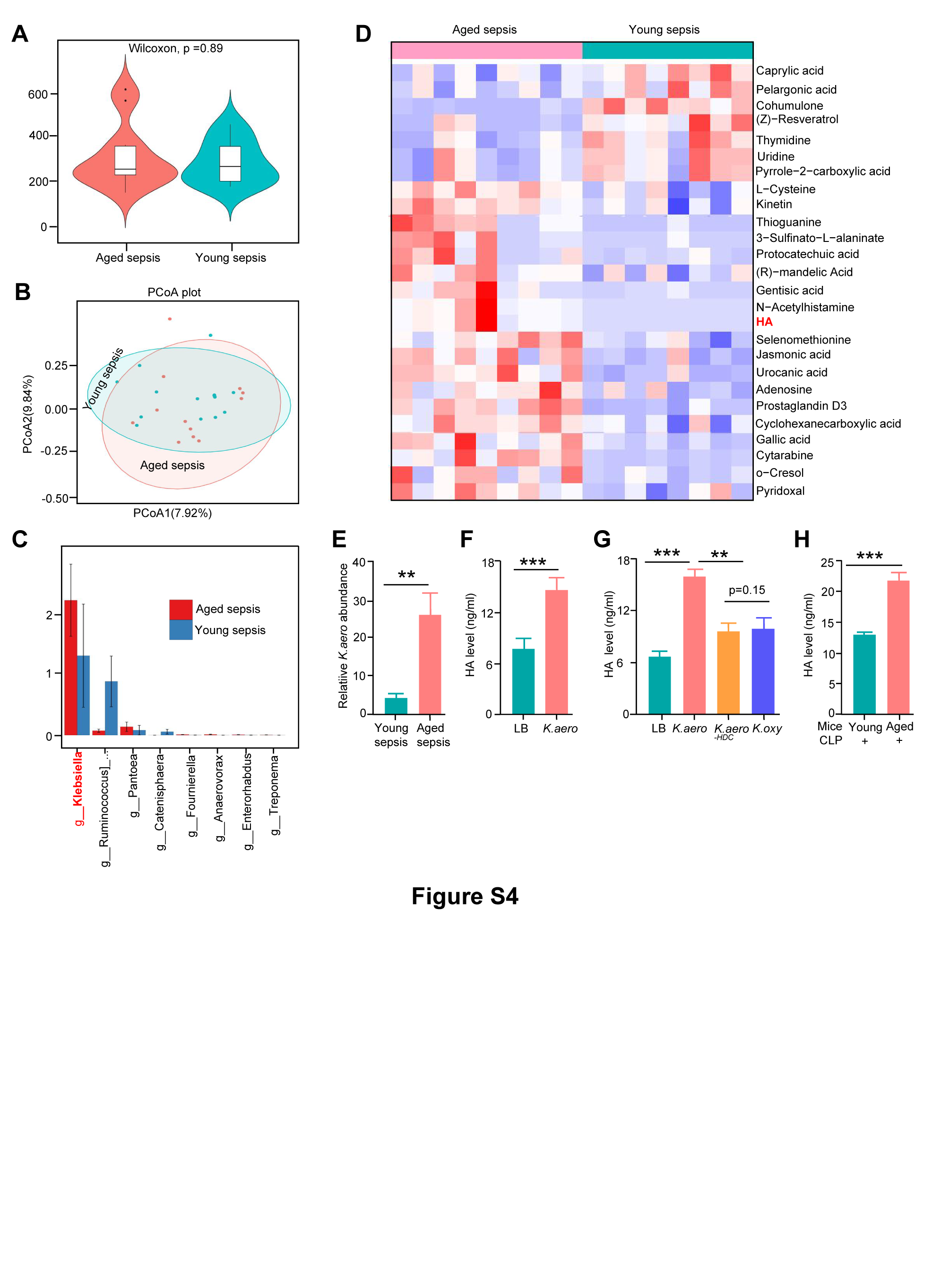


**Figure S3. Verification of gut microbiota colonization efficiency in FMT experiments**

(A-B) Alpha diversity(A) and beta-diversity(B) based on Chao1 and NMDS analysis for assessed the gut microbiota colonization efficiency of the fecal from the young and aged mice. AM, aged septic mice; YM, young septic mice; FMT-AM, the young mice recipients that received FMT from the aged septic mice; FMT-YM, the young mice recipients that received FMT from the young septic mice, n=12-13. ****, *p*<0.0001(C-D) Alpha diversity(C) and beta-diversity(D) based on Chao1 and NMDS analysis for assessed the gut microbiota colonization efficiency of the fecal from the young and aged mice. AP, aged septic patients; YP, young septic patients; FMT-AP, the young mice recipients that received FMT from the aged septic patients; FMT-YP, the young mice recipients that received FMT from the young septic patients, n=12-14.


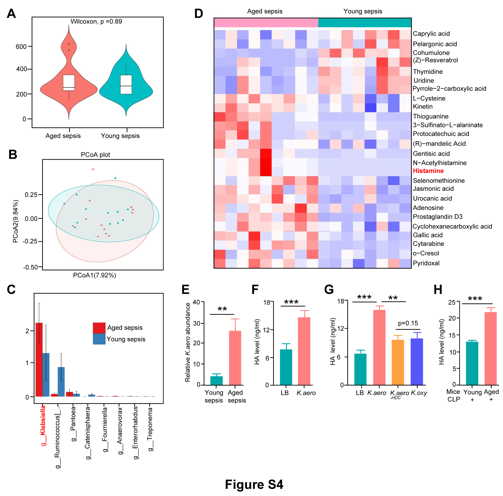


**Figure S4. The level of *K.aero* and metabolite HA is significantly increased in aged septic mice.**

(A) Alpha diversity based on Chao1 in young and aged septic patients. Young patients, n = 13; aged patients, n = 12.(B) PCoA using Bray-Curtis distance in young and aged septic patients. n = 12-13.(C) Relative abundance of gut microbiota at the genus level from the young and aged septic patients. young patients, n = 13; aged patients, n = 12.(D)Metabolomics analysis of cecum content from young and aged septic mice. Heatmap for metabolites were performed by the R package ggplot. n = 8-9. (E) Relative abundance of *K.aero* in septic aged and young patients. n = 16-18. **, *p*<0.01.(F) HA in culture supernatants of LB, wild-type *K. aero* strain. n=3. ***, *p*<0.001.(G)HA in culture supernatants of LB, wild-type, *K. oxy*, or *HDC*-deficient mutant of *K. aero* strain.n=3. **, *p*<0.01; ***, *p*<0.001.(H)HA concentration of aged and young septic mice. n = 11. ***, *p*<0.001.


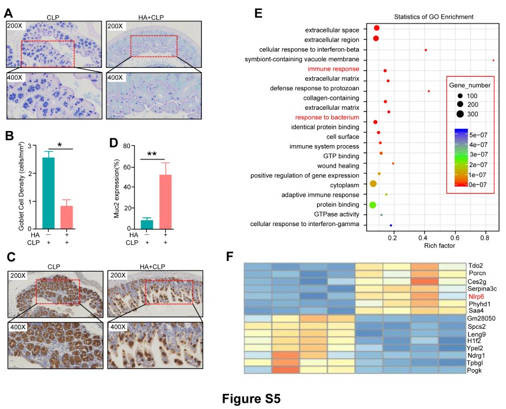


**Figure S5.** **HA modulates colonic goblet cells, Muc2, and age-related transcriptome in septic mice**

(A-B)AB-PAS staining (A) and goblet cell number (B) of colon from young septic mice pretreatment with or without HA. n = 4. *, *p*<0.05.(C-D)Immunohistochemistry (C) and quantification of Muc2 expression (D) of colon from young septic mice pretreatment with or without HA. n = 4. **, *p*<0.01.(E-F) Transcriptomic analysis of colon from young and aged septic mice. Statistics of GO enrichment analysis of differentially expressed genes were performed(E). Heatmap of differential gene expression(F). n = 4.


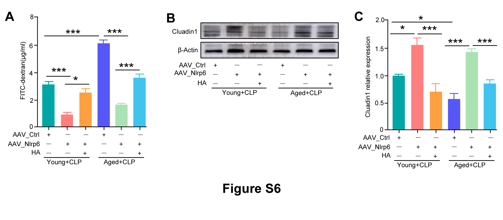


**Figure S6. HA may exacerbate intestinal barrier dysfunction by inhibiting the Nlrp6 expression in septic mice**

 (A) The serum FITC levels from the young and aged septic mice treated with HA after pretreated with Nlrp6 overexpression. n = 4. *, *p*<0.05; ***, *p*<0.001.(B) Western blot analysis for the quantification of protein expression of colon from young and aged septic mice. n =5.(C) Semi-quantitative analysis results of proteins of colon from young and aged septic mice. n =5. *, *p*<0.05; ***, *p*<0.001.


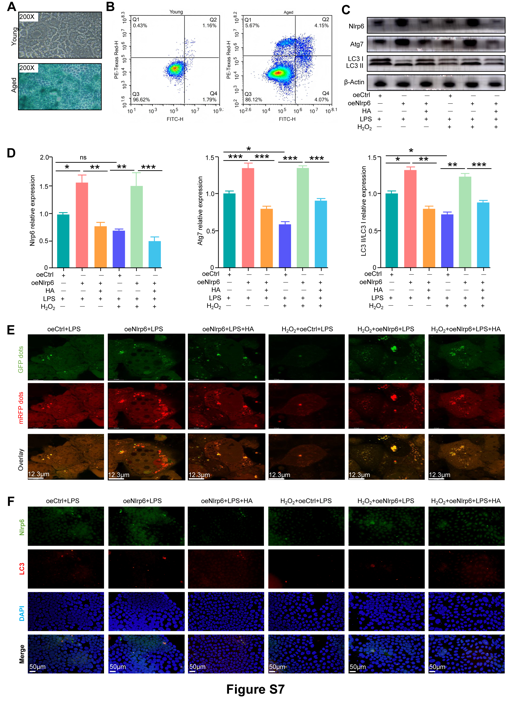


**Figure S7. HA-mediated regulation of Nlrp6 expression on autophagy in Caco2 cells.**

(A-B) β-galactosidase (A) and flow cytometry assay(B) to detect the senescence of Caco2 cells. n = 3.(D) (C-D)Western blot analysis for the quantification of proteins expression from young and aged LPS-treated Caco2 cells subjected to pretreatment with HA after the pcDNA 3.1 Nlrp6 or empty vector transfection. n = 3. ns,*p*>0.05; *, *p*<0.05; **, *p*<0.01; ***, *p*<0.001.(E)The autophagic flux level in young and aged LPS-treated Caco2 cells subjected to pretreatment with HA after the pcDNA 3.1 Nlrp6 or empty vector transfection. n = 3.(F) Immunofluorescence analysis for the co-localization of Nlrp6 and LC3 in young and aged LPS-treated Caco2 cells subjected to pretreatment with HA after the pcDNA 3.1 Nlrp6 or empty vector transfection. n = 3.


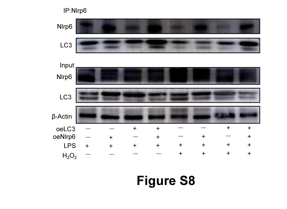


**Figure S8.** Co-IP assay showed that the binding between LC3 and Nlrp6 was reduced in senescent Caco2 cells. n = 3.
